# Complete Genome Sequence of vB_BveP-Goe6, a Virus Infecting *Bacillus velezensis* FZB42

**DOI:** 10.1128/genomeA.00008-18

**Published:** 2018-02-22

**Authors:** Tobias Schilling, Michael Hoppert, Rolf Daniel, Robert Hertel

**Affiliations:** a Genomic and Applied Microbiology & Göttingen Genomics Laboratory, Institute of Microbiology and Genetics, Georg-August University of Göttingen, Göttingen, Germany; b Department of General Microbiology, Institute of Microbiology and Genetics, Georg-August University of Göttingen, Göttingen, Germany

## Abstract

The new virus vB_BveP-Goe6 was isolated on the host organism *Bacillus velezensis* FZB42. The virus morphology indicated its association with the genus *Phi29virus*. The genome of vB_BveP-Goe6 (19,105 bp) comprises a linear chromosome with a GC content of 39.99%. The genome harbors 26 putative protein-coding genes and a noncoding packaging RNA.

## GENOME ANNOUNCEMENT

Viruses infecting bacteria are the most abundant biological entities on earth (1) and are ubiquitous in nature (2). Here, the complete genome sequence of the new virus isolate vB_BveP-Goe6 is reported. The naming of the isolate vB_BveP-Goe6 is based on the systematic schema suggested by Kropinski et al. (3). The virus vB_BveP-Goe6 was isolated from the Göttingen municipal sewage plant (Göttingen, Germany, 51°33ʹ15.4ʺN, 9°55ʹ06.4ʺE) via an overlay plaque assay using *Bacillus velezensis* FZB42 (4, 5) as a host.

Negative-staining transmission electron microscopy of virus particles showed a virion with head tail morphology, typical of the *Caudovirales* family. The short noncontractive tail revealed that vB_BveP-Goe6 is a member of the *Podoviridae* family. Virion dimensions of about 50 nm in width and 80 nm in length, together with the specific morphology of its tail, allowed further classification to the *Picovirinae* subfamily and indicated a relationship to the genus *Phi29virus* (6).

Viral genomic DNA was prepared with the MasterPure complete DNA and RNA purification kit (Epicentre, Madison, WI, USA). Paired-end Illumina sequencing libraries were generated with the Nextera XT DNA sample preparation kit and were sequenced with a MiSeq instrument and MiSeq reagent kit version 3 (Illumina, San Diego, CA, USA), as recommended by the manufacturer. Trimming and quality filtering of the recovered reads were performed with Trimmomatic version 0.36 (7) and analyzed with FastQC version 0.11.5 (8). The initial assembly was performed with SPAdes version 3.9.0 (9), resulting in a single contig with 1,000-fold coverage. Genome ends were verified via Sanger sequencing (10) and resulted in a final 19,105-bp linear chromosome with a GC content of 39.99%. BLASTn analysis against the nonredundant database of NCBI identified phi29 (GenBank accession no. NC_011048) as the closest relative, with 95% genome sequence identity. Additionally, a significant sequence identity of the vB_BveP-Goe6 genome was also recorded with genomes derived from other members of the *Phi29virus* genus, such as the viruses PZA (GenBank accession no. M11813), Nf (GenBank accession no. EU622808), B103 (GenBank accession no. NC_004165), and vB_BsuP-Goe1 (GenBank accession no. KU831549) (11). The identified inverted terminal repeats (5ʹ-AAAGTA) of vB_BveP-Goe6 perfectly matched the corresponding ones of other genus members, except virus GA-1 (GenBank accession no. NC_002649). Genome analysis and annotation resulted in the identification of 26 protein-coding genes, of which 18 were with predicted function and 8 were hypothetical proteins. Furthermore, a noncoding RNA could be identified. The annotated genes covered all functions necessary for the replication of a *Phi29virus* genus member (6). Direct comparison with the genome of phi29 (GenBank accession no. NC_011048) revealed that the genome of vB_BveP-Goe6 is 177 bp smaller. This difference is mainly due to the loss of a hypothetical protein gene located on the left early gene region of vB_BveP-Goe6. The noncoding packaging RNA (pRNA) was identified via a covariance model, which was created with the Infernal 1.1.2 software package (12) using the pRNA genes of virus Nf (GenBank accession no. EU622808) and GA-1 (GenBank accession no. NC_002649) as input sequences. The pRNA is part of the molecular motor, which is responsible for the genome translocation into the prohead.

### Accession number(s).

The genome sequence of vB_BveP-Goe6 was deposited in DDBJ/EMBL/GenBank under the accession no. MF407276.

## References

[1] Fuhrman JA 1999 Marine viruses and their biogeochemical and ecological effects. Nature 399:541–548. doi:10.1038/21119. 10376593

[2] Clokie MR, Millard AD, Letarov AV, Heaphy S 2011 Phages in nature. Bacteriophage 1:31–45. doi:10.4161/bact.1.1.14942. 21687533 PMC3109452

[3] Kropinski AM, Prangishvili D, Lavigne R 2009 Position paper: the creation of a rational scheme for the nomenclature of viruses of *Bacteria* and *Archaea*. Environ Microbiol 11:2775–2777. doi:10.1111/j.1462-2920.2009.01970.x. 19519870

[4] Chen XH, Koumoutsi A, Scholz R, Eisenreich A, Schneider K, Heinemeyer I, Morgenstern B, Voss B, Hess WR, Reva O, Junge H, Voigt B, Jungblut PR, Vater J, Süssmuth R, Liesegang H, Strittmatter A, Gottschalk G, Borriss R 2007 Comparative analysis of the complete genome sequence of the plant growth-promoting bacterium *Bacillus amyloliquefaciens* FZB42. Nat Biotechnol 25:1007–1014. doi:10.1038/nbt1325. 17704766

[5] Dunlap CA, Kim S-J, Kwon S-W, Rooney AP 2016 *Bacillus velezensis* is not a later heterotypic synonym of *Bacillus amyloliquefaciens*; *Bacillus methylotrophicus*, *Bacillus amyloliquefaciens* subsp. *plantarum* and “*Bacillus oryzicola*” are later heterotypic synonyms of *Bacillus velezensis* based on phylogenome. Int J Syst Evol Microbiol 66:1212–1217. doi:10.1099/ijsem.0.000858. 26702995

[6] Meijer WJJ, Horcajadas JA, Salas M 2001 29 family of phages. Microbiol Mol Biol Rev 65:261–287. doi:10.1128/MMBR.65.2.261-287.2001. 11381102 PMC99027

[7] Bolger AM, Lohse M, Usadel B 2014 Trimmomatic: a flexible trimmer for Illumina sequence data. Bioinformatics 30:2114–2120. doi:10.1093/bioinformatics/btu170. 24695404 PMC4103590

[8] Andrews S 2010 FastQC: a quality control tool for high throughput sequence data. https://www.bioinformatics.babraham.ac.uk/projects/fastqc/.

[9] Bankevich A, Nurk S, Antipov D, Gurevich AA, Dvorkin M, Kulikov AS, Lesin VM, Nikolenko SI, Pham S, Prjibelski AD, Pyshkin AV, Sirotkin AV, Vyahhi N, Tesler G, Alekseyev MA, Pevzner PA 2012 SPAdes: a new genome assembly algorithm and its applications to single-cell sequencing. J Comput Biol 19:455–477. doi:10.1089/cmb.2012.0021. 22506599 PMC3342519

[10] Willms I, Hoppert M, Hertel R 2017 Characterization of *Bacillus subtilis* viruses vB_BsuM-Goe2 and vB_BsuM-Goe3. Viruses 9:146. doi:10.3390/v9060146. PMC5490822 28604650

[11] Willms IM, Hertel R 2016 Phage vB_BsuP-Goe1: the smallest identified lytic phage of *Bacillus subtilis*. FEMS Microbiol Lett 363:fnw208. doi:10.1093/femsle/fnw208. 27609230

[12] Nawrocki EP, Eddy SR 2013 Infernal 1.1: 100-fold faster RNA homology searches. Bioinformatics 29:2933–2935. doi:10.1093/bioinformatics/btt509. 24008419 PMC3810854

